# Mechanical and Chemical Characterisation of TiN and AlTiSiN Coatings on a LPBF Processed IN718 Substrate

**DOI:** 10.3390/ma14164626

**Published:** 2021-08-17

**Authors:** Juan C. Colombo-Pulgarín, Antonio J. Sánchez Egea, Diego J. Celentano, Daniel Martínez Krahmer, Vitaliy Martynenko, Norberto López de Lacalle

**Affiliations:** 1 Department of Mechanical and Metallurgical Engineering, Engineering School, Pontificia Universidad Católica de Chile, Macul 7820436, Santiago, Chile; dcelentano@ing.puc.cl; 2 Politecnico di Milano, Chemistry, Materials and Chemistry Engineering Department, Piazza Leonardo da Vinci 32, 20133 Milan, Italy; 3 CNR ICMATE, National Research Council, Institute of Condensed Matter Chemistry and Technologies for Energy, Unit of Lecco, Via Previati 1/E, 23900 Lecco, Italy; 4 Department of Mechanical Engineering, Polytechnic University of Catalonia, C. Jordi Girona, 1-3, 08034 Barcelona, Spain; sanchezegea.antonio@gmail.com; 5 Research Center of Nanotechnology and Advanced Materials (CIEN-UC), Pontificia Universidad Católica de Chile, Av. Vicuña Mackenna 4860, Macul 7820436, Santiago, Chile; 6 Machining Processes and Metal Forming Department, National Institute of Industrial Technology (INTI), Av. Gral. Paz 5445, San Martín 1650, Buenos Aires, Argentina; dmartinez@inti.gob.ar (D.M.K.); vmartynenko@inti.gob.ar (V.M.); 7 Faculty of Engineering, National University of Lomas de Zamora, Lomas de Zamora 1832, Buenos Aires, Argentina; 8 Department of Mechanical Engineering, Aeronautics Advanced Manufacturing Center (CFAA), Faculty of Engineering of Bilbao, University of the Basque Country, Alameda de Urquijo s/n, 48013 Bilbao, Spain; norberto.lzlacalle@ehu.es

**Keywords:** TiN, LPBF, PVD coatings, roughness, wear, microhardness, GDOES, surface integrity, mechanical characterisation

## Abstract

Wear-resistant coatings development is progressively increasing steeply due to their advantages when applied to mechanical components subjected to abrasive and destructive environments. Titanium nitride (TiN) coating is typically used to enlarge tools and components’ service life and improve their surface quality. On the other hand, AlTiSiN coating intends to be applied to more aggressive environments such as spatial satellites components exposed to solar radiation, extremely high temperatures, and random particles impact. In this work, specimens of Inconel 718 (IN718) were fabricated via laser powder bed fusion (LPBF), and physical vapour deposition (PVD)-deposited with TiN and AlTiSiN as coatings to mechanically and chemically characterise their surface. In this respect, microhardness testing and chemical analysis via glow discharge optical emission spectroscopy (GDOES) were performed. Later, roughness and wear behaviour analyses were carried out to evaluate the mechanical performance of both coatings and their surface and morphological features. The experimental observations allowed the analysis of both studied coatings by comparing them with the substrate processed via LPBF.

## 1. Introduction

Coatings based on TiN became common in the coating of cutting tools in 1970 [1,2], since this material allowed for more durable parts. These coatings are the most frequently used because they combine features such as excellent resistance to wear, corrosion, and erosion. High bond strength to the substrate is also among their outstanding functional features [3,4]. Nowadays, materials such as steel for tools [5] and hard metals [6] need to work in aggressive environments where wear and corrosion are the main issues to be overcome. Consequently, surface coating technology has boomed in the last decade with two clear objectives: enhancing the decorative and/or functional aspects [7,8]. Decorative coatings are widely used in the jewellery sector to cover the substrate made of a much cheaper material, where sterling silver or gold are the most common coating. Simultaneously, copper, nickel, and stainless steel are less used due to allergic reactions [9,10]. Functional coatings are used to enhance the surface capabilities of the substrate, such as corrosion resistance, wear resistance, and lifespan, among others. However, the application of coatings on surfaces brings many challenges: obtaining a uniform surface quality by controlling the coating thickness, minimising the surface roughness [7], and reducing the cost of the product and enhancing the lifespan [11]. Thin hard coatings have been observed to protect against scratches without affecting material coatings. In ceramic or carbon coatings, the corrosion resistance can be improved in certain aggressive media [4].

Several coating techniques are often utilised to ensure a precision surface coating, such as physical vapour deposition (PVD) and chemical vapour deposition (CVD) techniques [12]. These techniques allow for making relatively thin films that bring enormous potential in coating complex specimens manufactured by laser powder bed fusion (LPBF) [13], dealing with intricate geometries with internal chambers and channels [14]. PVD and CVD are processes deposited from the vapour.

In many cases, high compressive stresses are an unwanted side effect of PVD coatings because they reduce the adhesive strength of the coating on the substrate [15,16]. However, in some applications, the PVD coatings primary focus consists of bringing the surface of the substrate into a compressive state. CVD is the deposition of a solid coating on a heated surface resulting from chemical reactions at the surface involving the surrounding vapour or gas. Typical CVD reactions include thermal decomposition, carburisation, and nitridation. These processes usually operate at temperatures over 850 °C. While cemented carbides (commonly coated by CVD for cutting tools) are not significantly affected by the processing temperature, the steels require additional heat treatment following coating to optimise their properties [1,17].

High demand for coatings is found in cutting and forming tools, such as drills, mills, broaches, dies moulds, and drawing dies [1]. Initially, the hard coatings are utilised in the cutting tool edge to improve the life cycle of a cutting tool when using the same operational parameters [18]. However, titanium nitride coatings (TiN, TiAlN) have shown to be an effective solution in other tooling issues such a machined material adhesion to the flutes and cutting tip, tool friction and build-up layer and edge. For example, a recent study [19] denoted that the presence of nanoparticles provides a high value of average microhardness and excellent performance in wear upon showing small minor surface scratches. Ni/TiN–SiC nanocoating showed a fine and uniform microstructure with large amounts of TiN and SiC nanoparticles at specific processing conditions of deposition. Only minor scratches were observed on the nanocoating surface. The same nanocoating deposited at different current densities showed different average microhardness, which evidenced the influence of the flux current.

Inconel 718 is a high-performance nickel-based alloy. This superalloy is broadly used for highly demanding engineering applications in aeronautical and aerospace industries due to its outstanding mechanical properties [20]. High-cost components are fabricated via LPBF, and their service life is interesting to be enlarged as much as possible. This can be done by coating them with micro and nano coatings such as TiN and AlTiSiN, making them interesting for the current study.

The work of [21] revealed directional independence of microhardness of samples in the as-built and heat-treated conditions. It also showed good values of tensile strengths and ductility compared to wrought IN718. Several coating strategies modify the substrate surface by using multi, simple, or nanolayers and gradient films or nanocomposites. A previous study [22] reported that the addition of SiC particles to a Ni–P metal matrix reduces the residual stress of the deposits and therefore eliminates the probability of surface cracking. SiC particles in the Ni–P–SiC deposit are not effective in dispersion hardening. A positive effect is also denoted in the microhardness of the composite as the mass fraction of P is reduced.

Conversely, the work of [23] reported significant changes in wear resistance when adding SiC particles to a Ni–P matrix. A fine microstructure was found to be positively correlated with microhardness. In the work of [24], it was established a surface roughness increasing because of the particle size increasing in reused powders during the printing process. Directly, the flowability of powder is also affected upon depositing a new layer. This work aimed to analyse the PVD coatings adhesion of specimens manufactured by laser powder bed fusion (LPBF).

Unlike the earlier studies, our objective is to study the surface quality and properties when using different coatings with the PVD technique in Inconel 718 specimens manufactured by metal additive manufacturing. Then, test specimens were produced and characterised by microhardness testing, chemical analysis via glow discharge optical emission spectroscopy (GDOES), surface roughness, and surface wear behaviour analysis to evaluate the mechanical performance of both coatings and their surface and morphological features.

## 2. Materials and Methods

### 2.1. Materials and LPBF Samples

**Figure 1:**
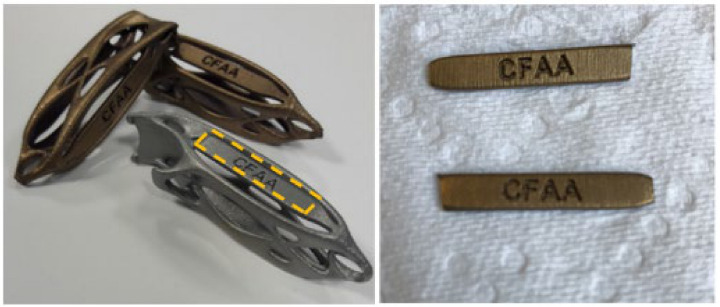
AlTiSiN coated specimens—Set 1.

**Figure 2:**
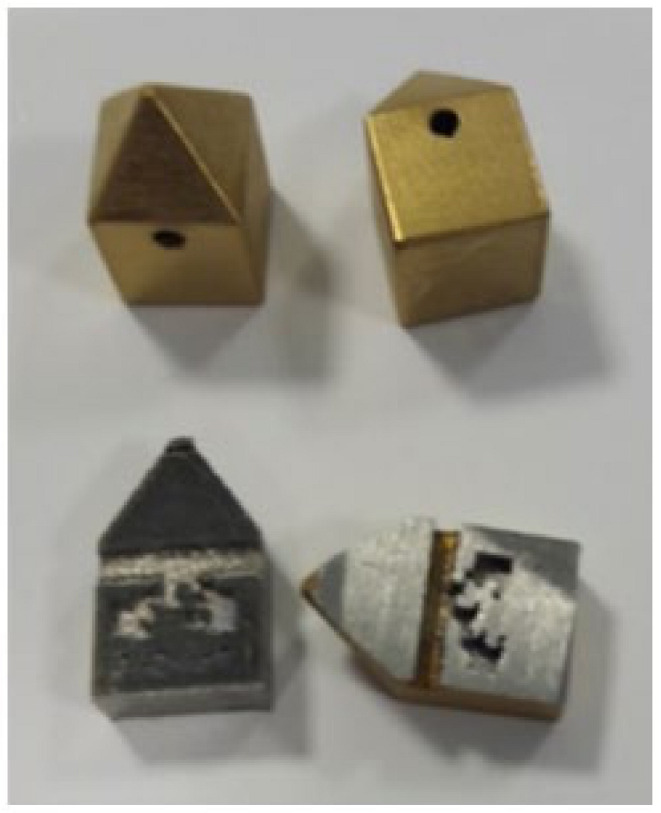
TiN-coated specimens—Set 2.

**Figure 3:**
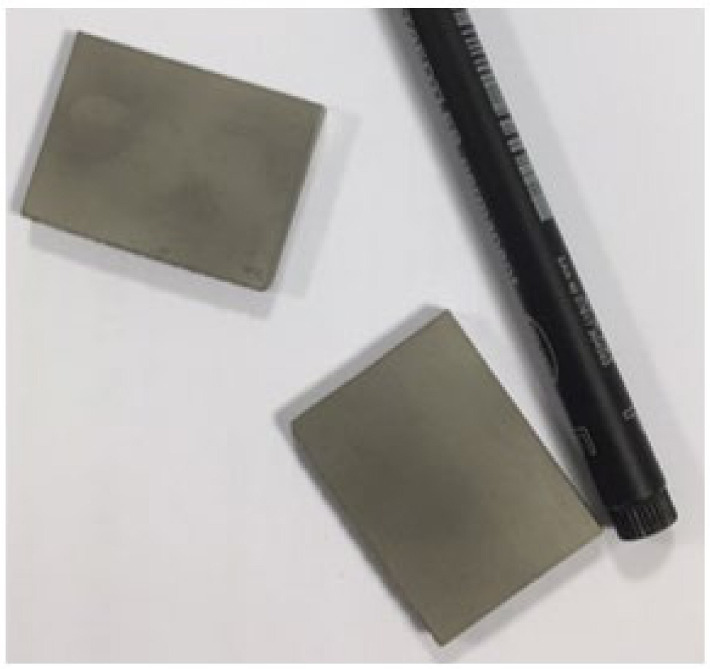
AlTiSiN coated specimens—Set 3.

Inconel 718 (CNPC Powder North America, Vancouver, Canada) was investigated in the present study as a substrate to produce three sets of specimens to evaluate the two coatings (Figure 1, Figure 2 and Figure 3). Sets 1 and 3 are AlTiSiN coated. Set 2 is TiN coated. The samples for all analyses were as follows.

Set 1 comprises two plates 20 mm × 4 mm × 2 mm (yellow highlighted in Figure 1), which were removed from the printed component via cutting process with a diamond disc machine. In this set, each plate has an outer face (OF) and inner face (IF).

Set 2 comprises three samples of 10 mm × 10 mm × 18 mm coated with TiN on all their faces (Figure 2).

Set 3 comprises two flat samples 40 mm × 30 mm × 5 mm coated with AlTiSiN on one of their larger areas faces each (Figure 3). In this set, each of the two plates has one coated surface and one uncoated surface.

The samples produced via LPBF were built with a Renishaw AM400 (Renishaw, Wotton-under-Edge, UK) system. This system uses a high stability Ytterbium fibre laser-focused and guided through a dedicated optical module to deliver energy at intensities high enough to fuse the metallic particles. Fibre lasers can cover an extended range of wavelengths by simply doping the core with different active dopants. Fibre lasers can also be used as seeds to produce high-performance supercontinuum sources. Continuous-wave supercontinuum generation extending to the visible spectral region has been demonstrated by pumping photonic crystal fibres at 1.07 μm with a 400 W single-mode CW Ytterbium fibre laser (Trumpf, Ditzingen, Germany) [25].

The AM400 system also uses a two-axis galvanometer to position the laser beam precisely in the X and Y axes. The optical system is calibrated to deliver a positioning accuracy of ±25 μm across the working area. The working volume sizes 250 mm × 250 mm × 300 mm for the X, Y, Z-axis respectively, whereas the nominal laser power is 400 W and the laser’s focal spot size is 70 μm.

The processing parameters used for printing the samples were: like-meander scanning strategy of 10 mm width stripes with 67° rotation between subsequent layers, 70 µm laser beam size, 40 µs exposure time, 30 µm layer thickness, 200 W laser power, and 35 µm hatch distance.

The manufacturing protocol for producing the samples via LPBF was the following. Firstly, the specimens’ CAD geometry was generated, converted into an STL file, and divided into layers of 30 microns of thickness. Subsequently, the additive strategy and process parameters were set up depending on the part material and its geometric features, such as internal features and contour zones. Later, the powder deposition, the laser spot guided by a galvanometric head, and the scanner melt at the part zone with a stripe trajectory were cyclically performed. Once a layer is processed, the substrate is moved down a distance equal to the previously set-up layer thickness. Another new layer of powder is deposited and extended by the tracker to onset the additive manufacturing process of a subsequent layer.

**Figure 4:**
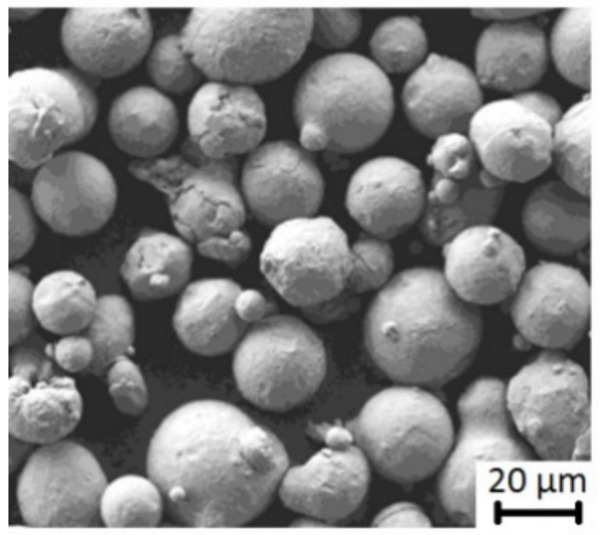
SEM images of the Inconel 718 powder after sieving.

The powder material used was Nickel Alloy Powder (REN-IN718) (CNPC Powder North America, Vancouver, Canada) with a particle size between 15 and 45 µm, as in the results of [26]. A SEM image of the powder material is shown in Figure 4.

The nominal chemical composition (wt.%) of the commercial Inconel 718 is shown in Table 1.

### 2.2. Coating Process of Samples

The PVD technology was used for the coating deposition on all specimens of both samples sets in the study. PVD process is widespread on the surface treatments of tooling components and tooling systems. This process involves the coating deposition on an atom-by-atom basis from the vapour phase, producing the vapour flux through a physical process (evaporation or sputtering). Once the coating flux encounters the component, it condenses, and single atoms are incorporated to form the coating [1,17]. Therefore, a single coating layer was applied for the TiN coated specimens, whereas a nanocoating strategy was selected for the AlTiSiN coated specimens. In the coating deposition process, the samples were placed on rotary plates during the whole coating cycle. The time of each cycle is 7–8 h, and the temperature was in the range interval 400–600 °C. This was done to guarantee the homogeneity of the coating deposition on all surfaces of the specimens.

### 2.3. Methods

#### 2.3.1. Microhardness

Microhardness measurements were taken with a Leco M400H (St. Joseph, MI, USA) hardness tester machine, using a Vickers nanoindenter (Wrexham, UK). All measurements were performed in the samples of sets 1 and 2 with 20 s of dwell time and are given in HV. The microhardness data was taken using a load of 100 grf [27]. Samples of set 3 were not used for microhardness measurements.

**Figure 5:**
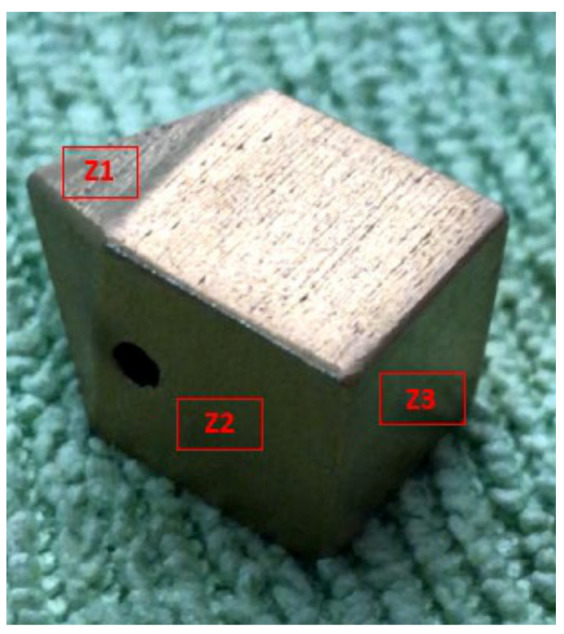
Zones of study on the representative sample of set 2. Z1—top (4 faces), Z2—walls (4 faces), Z3—bottom (1 face).

The mechanical characterisation was performed through a microhardness analysis. A test campaign was performed on this characterisation using the two samples of set 1 (AlTiSiN) and one sample of set 2 (TiN). In the sample of set 2, three different regions of the TiN-coated specimens were chosen for the analysis (Z1, Z2, Z3), as shown in Figure 5. The coating’s morphological and surface features were expected to change for each region because of the nature of the deposition process, which randomly impacts particles on the different surfaces while the sample rotates cyclically.

**Figure 6:**
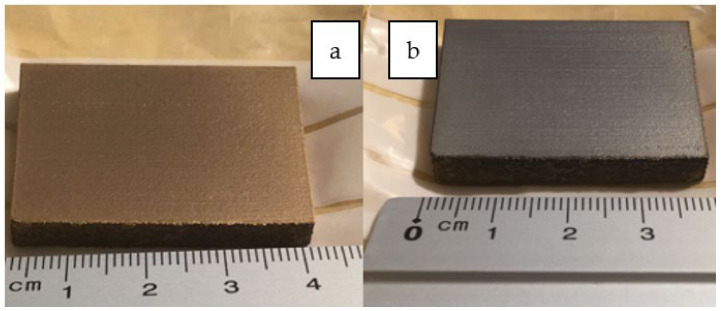
Zones of AlTiSiN samples of set 3: (**a**) coated surface and (**b**) uncoated surface.

As shown below in Figure 6a,b, two different regions of interest on the samples of set 3 coated with AlTiSiN were specified and differentiated: the coated surface and uncoated surface.

#### 2.3.2. Thickness

Coating thickness measurements were made by glow discharge optical emission spectroscopy (GDOES) analysis and Scanning Electron Microscope (SEM) using a Philips SEM 505 System (Eindhoven, The Netherlands). GDOES is an analytical technique used for material characterisation. Using this technique, a crater is made on the sample surface by sputtering with argon ions. This technique allows for obtaining the depth profile analysis of materials, simultaneously offering quantitative measurements of all constituents and thickness with nanometer depth resolution. During the GDOES analysis, three different measurements were performed at the same point but at different depths because of a restriction in the sampling area of the specimen.

Our study considered the analysis via GDOES to characterise the two PVD deposited coatings and the base metal. In the chemical study, a by-triplicate bulk analysis was done for determining the chemical composition of IN718. This analysis provided information about which elements were present in the specimen and the corresponding amount or mass concentration. Secondly, a DPA (Destructive Physical Analysis) and by-triplicate bulk analysis on both coatings were carried out. The DPA consisted of ion-bombarding the coated samples to obtain the surface/depth profile and the bulk elemental composition. As its name says, it physically destroys the bombarded area on the sample because of the impact of argon ions on the sample surface. Both coatings were also analysed via SEM.

#### 2.3.3. Roughness

**Figure 7:**
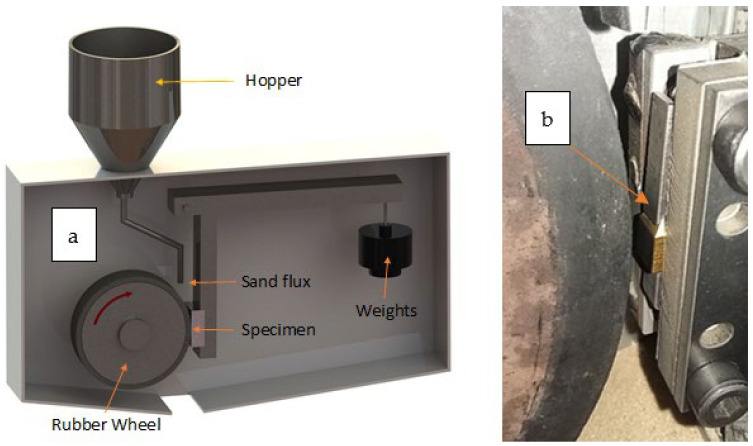
(**a**) Scheme of the machine for the wear tests and (**b**) clamping device to adapt set 2 samples to the machine.

The roughness surface parameters were determined using a Taylor Hobson Surtronic 3+ (Leicester, England) portable rugosimeter that used a 2.5 mm cut off and 12.5 mm sampling length. The wear behavior study was performed with a made-in-house machine (designed and built at the National Institute of Industrial Technology-INTI, San Martín-Buenos Aires, Argentina) following the ASTM G65 standard, which establishes the laboratory method to determine the relative abrasion resistance of various materials. The test involves rubbing a rectangular sample (usually, width and length of 25 mm × 70 mm and thickness between 3.2 mm and 12.7 mm) using river sand as an abrasive element (size between sieve meshes #50–#100). The sand is introduced between the vertically oriented rubber 230 mm diameter wheel rotating at a specific speed (200 rpm) and the specimen, which is held against the wheel at a specified normal weight load (the machine allows setting three different weight load values: 23 N, 45 N, and 130 N). The rotating rubber wheel pulls the sand into the contact area, rubbing the surface of the specimen. Wear rates are reported as weight loss. In this study, the controlled variables were: LPBF Inconel coated wear specimen, dry test, 45 N weight load for the AlTiSiN samples and 23 N weight load for the TiN samples, 200 rpm wheel rotation, rubber wheel, 360 g/min sand flow, and 180 s of test duration [28]. A schematic of the testing equipment is shown in Figure 7a. The experimental data of weights were obtained with Metler Toledo AB 204 (Greifensee, Switzerland) balance with 0.1 mg resolution.

#### 2.3.4. Wear

**Figure 8:**
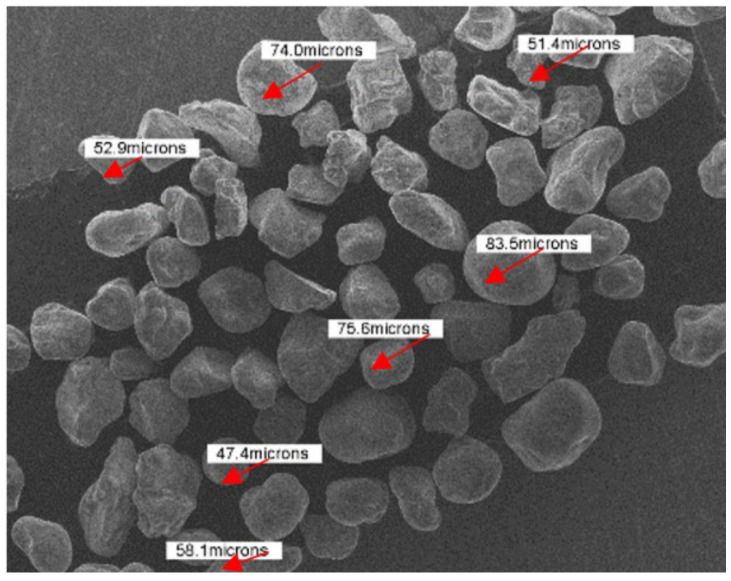
Morphology and dimension of abrasive sand.

The morphology and dimension of the sand used as an abrasive material in the wear behaviour study were established through SEM. The equivalent diameter of sand particles is 66 µm. Figure 8 shows a SEM image of the abrasive sand.

Initially, the wear analysis started with the samples of set 3. Considering the lack of available samples, the initial tests were performed with different metal sacrificial flat samples. The tests made with the sacrificial samples allowed calibrating the footprint placing and were centred considering the study area and allowed analysing the footprint as a function of load-time relationship. Based on the results of the initial tests, conclusions were sought to define the conditions to be set in the tests with the actual samples of set 3. For testing the TiN coated sample (set 2), a clamping device shown in Figure 7b was also designed and built to adapt it to the machine. The general parameters of the wear test are given in Table 2.

Based on the thickness of the coatings and considering the results of [4], the load value of 45 N was considered as the one for evaluating AlTiSiN (set 3) and 23 N for evaluating TiN in such a way that the contact pressure on the square-face of the sample of set 2 is similar to the contact pressure used with AlTiSiN.

## 3. Results and Discussion

### 3.1. Microhardness

**Figure 9:**
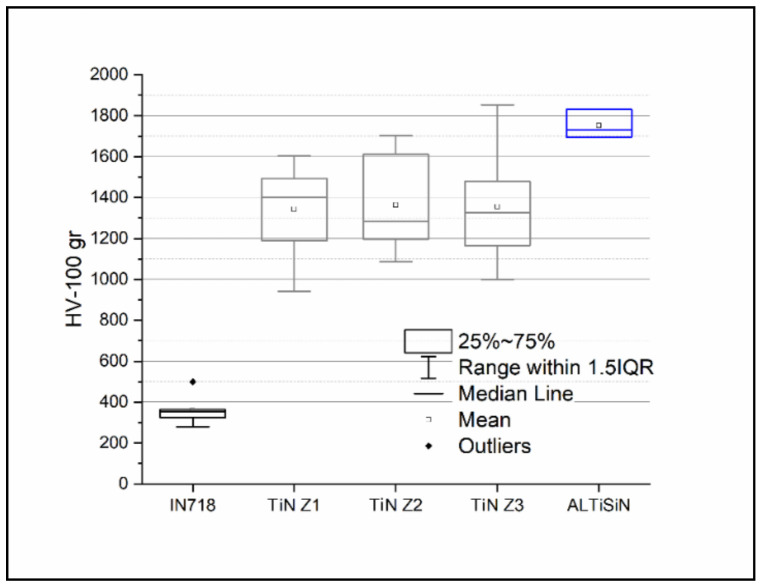
Vickers hardness for different evaluated surfaces.

The results of the different surfaces of the evaluated samples are shown in Figure 9.

From the obtained results, it may be stated that the hardness of the TiN coating deposited on IN718 is around 1355 HV, whereas the hardness of AlTiSiN coating deposited on IN718 is about 1750 HV. These values are higher than the hardness value of IN718, which is around 360 HV, as reported in [21]. Regarding specific values of the hardness of TiN coating, marked differences were not observed in the different evaluated zones (Z1, Z2, Z3).

### 3.2. Thickness

Good homogeneity features of the coatings are observed in their constitutive layers. Good adhesion and a low presence of defects may be inferred. The weight percentages of the alloy constituent elements are shown in Table 3.

**Figure 10:**
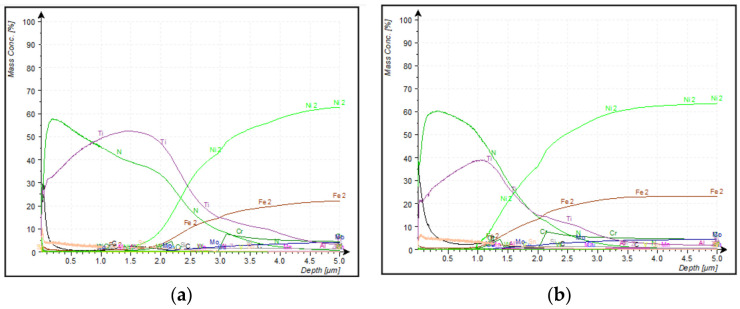
Percentage mass concentration vs. depth of TiN coating in (**a**) Z2 surface and (**b**) Z3 surface.

TiN thickness: The by-triplicate bulk analysis of each coating was also performed before DPA to determine which elements are present. The DPA was carried out in a second instance (upon knowing and selecting the found elements). The DPA on Z1 of the TiN coated specimen was not possible due to the geometrical disposition of the face compared to the direction of the sputtered electrons during the process. It does not allow for the specimen to be stabilised in the GDOES system. Figure 10 showed the variation of mass concentration of the constituent elements of the TiN coating as the depth increased.

Ti and N were the two most abundant elements found in the coating surface in zones Z2 and Z3 of the set 1 sample (Z2: 43.9 m% Ti and 41.5 m% N; Z3: 26.2 m% Ti and 36.9 m% N). As shown in the curves of Figure 10, their mass concentration started to decrease at different depths for both Z2 and Z3 as follows: in Z2, Ti mass concentration began to decline at around 1.5 µm depth, and N mass concentration started to decrease at approximately 0.3 µm depth. On the other hand, in Z3, Ti mass concentration declined at around 1.2 µm depth, and N mass concentration decreased at about 0.3 µm depth, as occurred in Z2. The presence of C was also detected in the coating surface. This is mainly due to some C migration from the substrate material, as it was reported to be present in the initial substrate composition.

At the depth at which Ti mass concentration started to decline (purple tendency line in both-zones/both-charts), the chemical concentration of the base metal main constituent (Ni) started to increase. The depth at which variation of concentrations of the main constituent of coating (decreasing of Ti) and the main constituent of base metal (increasing of Ni) occurred can be taken to indicate the coating thickness DPA results. However, the transition of the constituent composition curves is because the process is performed in the plasma state, so some remaining elements are kept during the measurements. Therefore, at depths on which curves intersect, the limit of coatings is placed.

It means that the coating thickness in Z2 and Z3 is about 2.5 µm and 1.5 µm, respectively. The thickness variation is due to the mechanism of deposition of the coating via PVD. During the PVD process, the impact direction in the sides of the Z2 allowed an inevitable accumulation of the coating material before consolidation, making the thickness in those faces higher than that of Z3.

Beyond 5 µm onwards, the composition curves behaviour of the remaining constituent elements was constant on both Z2 and Z3 zones, so it can be understood that base metal was reached.

AlTiSiN thickness: Ti and Si were the two most abundant constituents in AlTiSiN coating at both faces (OF and IF). It is reported in Table 4.

**Figure 11:**
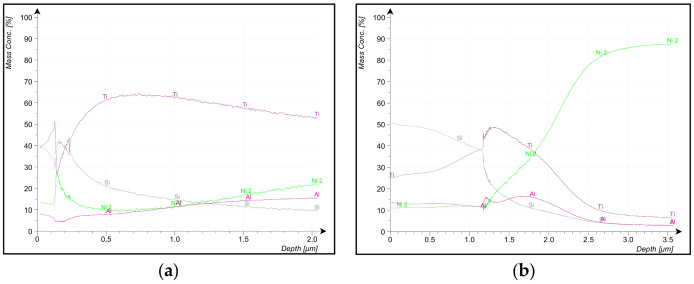
Percentage of mass concentration vs. depth of AlTiSiN coating in (**a**) outer face and (**b**) inner face.

Results of DPA of AlTiSiN are shown in Figure 11. The thickness of AlTiSiN analysed from GDOES was done with the samples of set 1. The DPA results of the two samples showed the variation of mass concentration of the constituent elements of the coating as the depth increased. The Ti/Si ratio variation is because the thickness and composition are not the same in OF and IF. A similar ratio variation was observed with the TiN coating on Z2 and Z3.

Figure 11a,b show that Ti and Si mass concentration started to decrease at different depths for the inner face and outer face as follows. Ti mass concentration started to decline on the inner face at around 1.35 µm depth, and Si mass concentration decreased entirely from the coating surface. On the outer face, Ti mass concentration decreased at around 0.75 µm depth. Si mass concentration declined from the coating surface with a slight increase around 0.15 µm depth and continued to decrease continuously until stabilised in the metal base. Between 0.1 and 0.2 µm depth, peaks and drops of mass concentrations also state an inhomogeneity inside the coating in the vicinity of the analysis point. The coating thickness of the AlTiSiN coating on the inner face and the outer face is approximately 1.2 µm and 1.0 µm.

**Figure 12:**
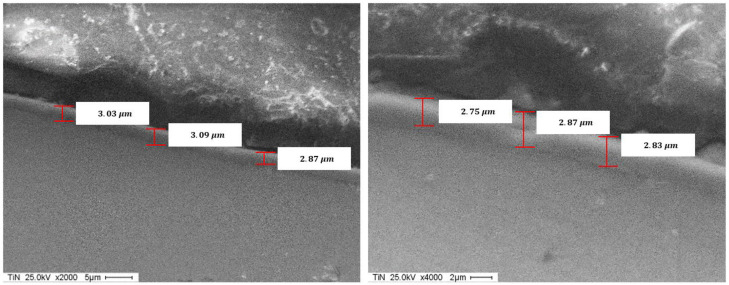
SEM of the TiN coating.

**Figure 13:**
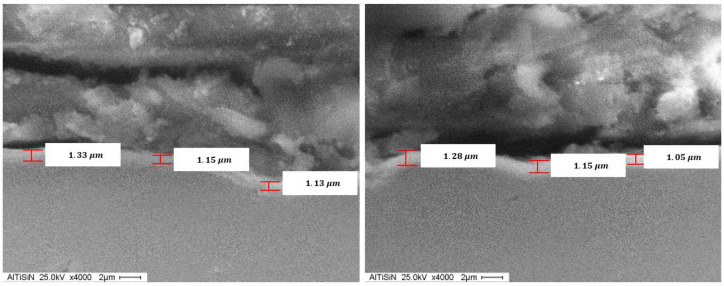
SEM of the AlTiSiN coating.

Figure 12 and Figure 13 show the SEM images of the TiN and AlTiSiN coatings. The thickness values in different points of the coatings can be observed.

Comparatively, the thickness measured values for both coatings obtained from the GDOES analysis and SEM analysis is reported in Table 5.

### 3.3. Roughness

The roughness study was done with samples of set 3 (coated and uncoated faces) and one sample of set 2. It defines three different sets of data.

The used rugosimeter provides six different roughness parameters, but the Rz values were considered because it is usually the roughness parameter used to compare samples produced via LPBF [26]. The measurements were taken in a perpendicular direction to the roughness stripes and reported in Table 6.

The roughness values between the uncoated and coated surfaces in the samples of set 3 (AlTiSiN coated) are not big enough. This is most likely due to small values of the coating thickness. On the contrary, the TiN roughness value is smaller compared to AlTiSiN. Depending on the direction of roughness stripes and overhanging angle, a correlation can be established, as mentioned by [20].

### 3.4. Wear

**Figure 14:**
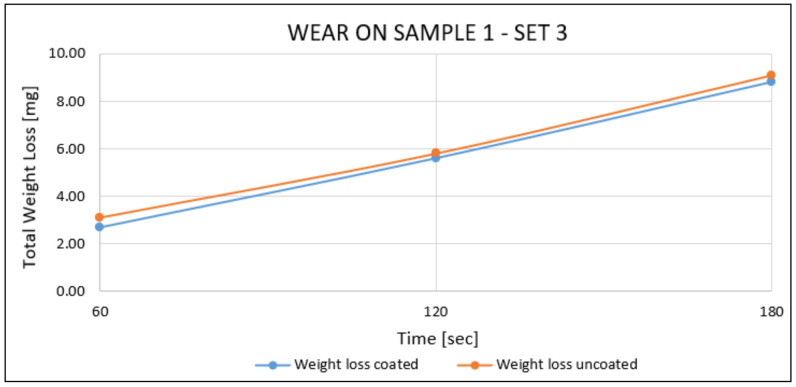
Wear results on the flat sample 1.

Sample 1—Set 3:

Based on the results of [4] and safety reasons, sample 1 of set 3 was tested with time steps of 60 s. The results on both surfaces of sample 1 of set 3 showed that AlTiSiN coating disappeared in a time value of less than 1 min. It can also be observed that the wear rate of the coated and uncoated surfaces was roughly the same. This behaviour can be observed in Figure 14.

Because of the results obtained with sample 1 of set 3, sample 2—of the same set—was tested with shorter time values.

**Figure 15:**
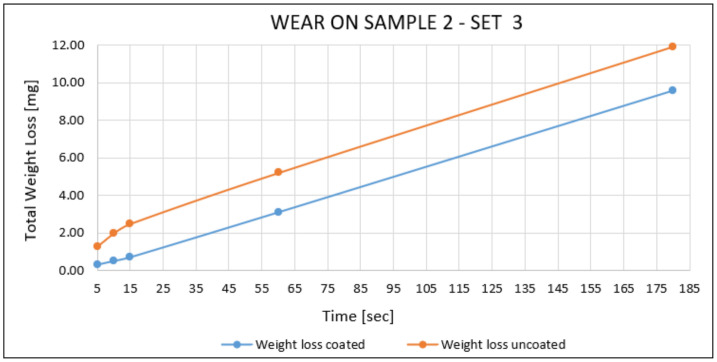
Wear results on the flat sample 2.

Sample 2—Set 3:

Wear results of sample 2 tested with 5 s, 10 s, 15 s, 60 s, and 180 s are shown below in Figure 15. An increasing tendency of total weight loss can be observed simultaneously as the contact time with the rubber wheel increased. Relatively to the substrate material, the wear resistance behaviour of the coating showed less weight loss at each test-time value.

**Figure 16:**
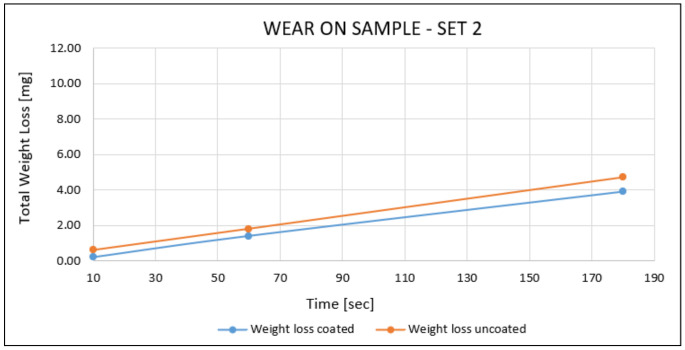
Wear results on the sample—set 2.

Sample—Set 2:

In this sample, the wear tests were performed on one of the squared faces of zone Z2 (Figure 5). As mentioned before, a test load of 23 N was defined to keep a similar contact pressure as the one used in the samples of set 3 with a test time of 180 s. Because of the higher thickness of this sample, the test-time values were 10 s, 60 s, and 180 s. Although the coatings showed a lower wear rate than the substrate, the difference was slight (no higher than 17%). The results are shown below in Figure 16.

## 4. Conclusions

Specimens of IN718 with TiN and AlTiSiN PVD deposited with PVD, ranging from 1 µm to 3 µm, approximately, were investigated by analysing their mechanical performance (microhardness and wear studies) and morphology (roughness study). Coating thicknesses were also determined and compared using two different techniques: GDOES analysis and SEM analysis. The main conclusions of the work are summarised as follow:

- The measured microhardness value on AlTiSiN and TiN was higher than the one of the substrate materials. Among them, the Vickers microhardness of the former is higher than the one of the latter. The microhardness measurement method is comparative.
- In the wear study, AlTiSiN showed better wear resistance as compared to TiN. By evaluating the same test-time values of both coatings with the rubber wheel (10 s, 60 s, and 180 s), there was found less relative weight loss in AlTiSiN by considering its respective weight loss of substrate material. The lower the relative weight loss, the better the mechanical adhesion of the coating to the substrate material.
- The roughness of AlTiSiN was found to be higher than the one of TiN. From the DPA results of the former, titanium accumulation was found to be present, so it may negatively affect the final quality of the surface and make it rougher.
- The thickness measurement results of both coatings were in good agreement by using two different measurement techniques. The thickness value of AlTiSiN was found to be smaller than the one of TiN. Despite this, its wear resistance was found to be better, as mentioned above.

## Figures and Tables

**Table 1:** Nominal chemical composition of commercial IN718.

Fe	Ni	Mo	Ti	Nb	Cr	Al	C	Si	Co
Balance	50–55	2.8–3–3	0.8–1.2	4.8–5-5	17–21	0.3–0.7	0.02–0.08	0.35–Max	1.00–Max

**Table 2:** General parameters of the wear test.

Rubber Wheel Diameter (*φ_RW_*) (mm)	Wheel Rotation (*ω*) (rpm)	Sand Flow (*ϕ_s_*) (gr/min)
230	200	360 ± 10

**Table 3:** Chemical composition of the substrate obtained via GDOES.

Elements	Ni (%)	Fe (%)	Cr 2 (%)	Co (%)	Mo (%)	C (%)	Mn (%)	Si (%)	(P, S, Cu, Al, Ti, V, Nb, W) (%)
Test 1	48.86	18.61	22.33	0.28	3.43	0.21	0.07	0.10	6.11
Test 2	50.66	18.24	20.81	0.27	3.48	0.15	0.08	0.10	6.21
Test 3	51.66	17.99	19.96	0.27	3.55	0.05	0.08	0.09	6.34
Average	50.39	18.28	21.03	0.27	3.49	0.13	0.08	0.10	6.22

**Table 4:** Average mass concentration of Ti and Si in AlTiSiN coated specimen.

E/T	Inner Face		Outer Face	
Elements	Ti (%)	Si (%)	Ti (%)	Si (%)
Test 1	42.5	21.3	56.7	12.4
Test 2	33.5	29.4	56.5	17.9
AV	38.0	25.4	56.6	15.2

**Table 5:** Values of thickness for both coatings.

GDOES				SEM		
TiN|Z2	TiN|Z3	AlTiSiN|IF	AlTiSiN|OF	AV/σ	TiN	AlTiSiN
2.5 µm	1.5 µm	1.2 µm	1.0 µm	**AV**	2.9 µm	1.2 µm
				**σ**	0.1	0.1

**Table 6:** Roughness values of samples (AV = Mean value; σ = standard deviation; COV = Coefficient of variation).

U/C	Sample 1 [AlTiSiN]—Set 3 Rz DIN			Sample 2 [AlTiSiN]—Set 3 Rz DIN			Sample [TiN]—Set 2 Rz DIN		
	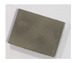			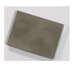			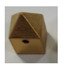		
	AV	σ	COV	AV	σ	COV	AV	σ	COV
**Uncoated**	23.0	3.7	15.9	30.3	3.4	11.2	-		
**Coated**	33.0	2.8	8.6	25.2	3.5	14.1	17.8	4.6	25.6

## Data Availability

Data sharing is not applicable.
